# Smoking history, eligibility for lung cancer screening and risk of death by lung cancer or other causes -- a longitudinal, time-variable analysis of the EPIC-Heidelberg cohort

**DOI:** 10.1186/s12916-026-04891-z

**Published:** 2026-04-28

**Authors:** Yue Chen, Srimanti Dutta, Rashmita Bajracharya, Francisco Cortès-Ibañez, Renée Turzanski Fortner, Verena Katzke, Rudolf Kaaks

**Affiliations:** 1 Division of Cancer Epidemiology, German Cancer Research Center (DKFZ), Heidelberg, Germany; 2 Translational Lung Research Center Heidelberg (TLRC-H), the German Center for Lung Research (DZL), Heidelberg, Germany; 3 Department of Research, Cancer Registry of Norway, Norwegian Institute of Public Health, Oslo, Norway

**Keywords:** Smoking history, Smoking cessation, Mortality, Lung cancer, Screening eligibility criteria

## Abstract

**Background:**

Eligibility criteria for lung cancer (LC) screening aim to identify long-term smokers who have exceeded a minimum-risk threshold of having LC, while still having sufficiently long remaining life expectancy. For individuals who have once met eligibility criteria (i.e., reached sufficiently high LC risk), a question is whether longer-term smoking cessation and corresponding improvement in other-cause mortality risk could justify a higher maximum screening age for former smokers than for continuing smokers.

**Methods:**

We performed time-varying Cox models in the EPIC-Heidelberg cohort (N = 24,715), using 3-yearly questionnaire data collected between 1994 and 2014 to estimate hazard ratios (HRs) and incidence rates for death by LC or other causes, in relation to age-specific smoking status and eligibility by German LC screening criteria (LCSC).

**Results:**

Depending on age, models showed up to 3-fold higher risk of other-cause mortality for recent and LCSC-eligible smokers, relative to never smokers. Among former smokers, those who quit before age 30 or 40 showed no significant difference in other-cause mortality compared with never-smokers, in men and women, respectively. However, at higher quitting ages HRs increased up to about 1.9 for men who stopped at age ≥60, or 1.5 in women who stopped at age 50-<60. Also, depending on age, former smokers who had once met the LCSC, but then quit for >10 years (thereby losing formal screening eligibility), showed HRs of about 1.5–3.0 for other-cause mortality in men and 1.3–1.9 in women. In both sexes, the absolute incidence rate for other-cause mortality amongst past-eligible smokers age 75-<80 was similar to that for current-eligible smokers of the younger, 70-<75-year age group. For LC, both current and former smoking were associated with persistently increased HRs, even after long-term cessation.

**Conclusions:**

For smokers who once met LCSC, but then quit for >10 years the risk of other-causes mortality remains elevated, but less so than for continuing smokers, which may argue for a moderate extension of the maximum age limit for LC screening. Larger studies will be needed to obtain more precise risk estimates.

## Background

Smoking is a leading cause of lung cancer (LC) development and mortality [1–8], but also increases early mortality by other causes [5, 7–11]. To optimize the efficacy of LC screening by low-dose computed tomography (LDCT), both the effects of lifetime smoking history on LC risk on the one hand, and risk of death by other causes on the other hand, should be accounted for [5, 7–11].

In the USA, Canada, the UK and several further European countries, LC screening by low-dose computed tomography (LDCT) has been, or is currently being, introduced [12–16]. In these countries, eligibility for screening is determined on the basis of age limits, in combination with an individual’s smoking history, usually defined by exceeding a given threshold of life-time smoking duration and/or cumulative exposure to cigarette smoke (e.g., in pack-years) plus a maximum time since quitting, for an ex-smoker [12–16]. These criteria aim to ensure that, on the one hand, individuals meet a minimum-risk threshold of having LC (which determines numbers that need to be screened to detect a LC case) while, on the other hand, the maximum age limit should help ensure that participants still have a sufficient remaining life expectancy. A sufficient remaining life expectancy is important, as it determines whether a screening participant may still gain a relevant number of life years through early detection and treatment of a lung tumor. Furthermore, a longer remaining life expectancy is associated with a lower risk of over-diagnosis – i.e., diagnosis of tumors that without screening would not have become clinically manifest, as a screening participant would have died before by another cause.

For individuals who meet the requirement of reaching a minimum risk threshold for LC, qualifying them to participate in LC screening, it is being questioned whether longer-term smoking cessation is a useful criterion to stop screening [2–4, 17–20], or whether, on the contrary, longer-term quitters who have once been eligible should not rather be granted a higher stopping age than screening participants who continued smoking. Large-scale population studies in the USA and the UK have shown that, at any given age, the excess risk for all-cause mortality (reduction in residual life expectancy) is greater for current smokers than for ex-smokers, depending on actual quitting times, and that longer-term quitting also diminishes the risk for all-cause mortality relative to never-smokers [5, 7–9, 21, 22].

To gain further information on the above questions, we performed a longitudinal, time-variable analysis of the EPIC-Heidelberg cohort (Germany), estimate HR for death by LC, other causes or all causes combined in relation to smoking status, eligibility status for screening according to official German criteria [13], and smoking cessation times.

## Methods

### EPIC-Heidelberg cohort

The EPIC-Heidelberg cohort comprises 25,546 men and women from the general population living in the southern German city of Heidelberg and its surrounding municipality at recruitment. The design and methods for the EPIC prospective cohort study have been described in detail previously [23, 24]. In brief, participants were recruited randomly between 1994 and 1998 from the population registries of Heidelberg and its surrounding areas, enrolling women aged 35 to 65 year and men aged 40–70 years at the time of first inclusion in the study.

Baseline examinations included measurement of anthropometric indices, and assessment of lifestyle factors and dietary habits via comprehensive questionnaires. Questionnaire data were also collected on lifetime history of tobacco smoking (smoking status, age at start, duration [years], average intensity [cigarettes/day], and time since quitting for ex-smokers [years]). For regular updates after baseline recruitment, individuals were asked at 3-year intervals complete follow-up questionnaires, for a total of 6 follow-up rounds through the end of 2014. Standardized core questions were asked at baseline and in each follow-up round, and in follow-up rounds 2–6, this additionally included questions about current smoking status and time at which a participant stopped smoking. Telephone contacts were used to maximize participation and completion rates for each of the follow-up questionnaires.

Incident cancer cases were prospectively ascertained by a combination of active follow-up (self-reports, with subsequent verification of clinical records) and linkages to regional cancer registries. Mortality endpoints were ascertained through regular linkages to municipal records (vital status), followed by information collection on causes of death from regional health offices. The present analyses were based on the mortality follow-up data until May 2019, covering up to five years of observation time after the last follow-up questionnaires administered in 2014.

### Calculation and imputation of smoking-related variables

From baseline and successive follow-up questionnaires, for each questionnaire date the following smoking-related variables were extracted or calculated: smoking status (recent, former, never), lifetime smoking duration (years), lifetime cessation duration (years), daily smoking consumption (cigarettes/day), cumulative lifetime pack-year exposure, and eligibility status according to the German LC screening criteria (LCSC). Eligibility by LCSC was categorized as never smokers, smokers who at a given time point were eligible [“current eligible”], smokers who have been eligible in the past [“past-eligible”] and smokers who never reached eligibility status [“never eligible” smokers]. Recent smokers were defined as participants who reported current smoking, or who reported having stopped cumulatively less than 2 years. The German criteria for LC screening eligibility are: being 50–75 years of age, having smoked at least for 25 years and at least 15 pack-years, less than 10 years’ time since quitting for ex-smokers.

Lifetime smoking duration and lifetime cessation duration were derived from participants’ self-reported smoking information and calculated only for those who responded to the respective questionnaire. For calculations the following assumptions were used: if smoking status remained unchanged between two consecutive follow-up rounds, participants were assumed to continue smoking or not smoking during that interval. If smoking status changed, duration was estimated based on the reported year of quitting or, if unavailable, using the time interval midpoint to allocate person-time between smoking and cessation. For daily smoking consumption (cigarettes/day), missing values were linearly interpolated when data were available at adjacent questionnaires. For smokers who never reported daily consumption at any round, multiple imputation was applied. Further details are in the appendix (Additional file 1: Method S1).

### Statistical analyses

For statistical analyses, study participants were excluded when: (1) smoking status was unknown at recruitment; (2) individuals had LC diagnosis prior to recruitment; or (3) survival status was unknown since baseline recruitment, leaving a total of 24,715 participants.

Basic characteristics of participants during each follow-up period were described using frequencies and percentages for categorical variables, and medians with inter-quartile ranges (IQR) for continuous variables.

Time-dependent Cox models were used to estimate the associations between smoking-related variables (regularly assessed through questionnaires, from baseline till latest follow-up) and mortality, using age as the underlying time scale, stratified by sex and without further covariate adjustments. Censoring was defined as follows: for participants permanently lost to follow-up, censoring occurred at the expected date of the next follow-up (6 years after baseline, or 3 years after the second to fifth follow-ups) or, for those who completed the last (sixth) follow-up, 5 years after that final questionnaire response. Deaths occurring before the censoring time were considered events; otherwise, observations were censored.

We modelled mortality risks for three types of exposure definition: (1) continuous variables for lifetime smoking duration (years), lifetime cessation duration (years), and smoking intensity (cigarettes/day); (2) smoking status (recent, former, never); and (3) eligibility according to the German LCSC (never, never eligible, current eligible, and past eligible smokers). Outcomes included LC-specific mortality, mortality from other causes, and all-cause mortality. For the model series (2) and (3), hazard ratios (HR) for mortality in relation to smoking status or screening eligibility were estimated within restricted (5- or 10-year) age windows.

## Results

### Cohort description: prospective evolution of smoking habits

At baseline, our analyses included 24,715 participants (11,594 men, 13,121 women), with median age of 51.0 years (interquartile range [IQR]: 44.0–58.0) and comprising 41.9% never smokers, 33.0% former smokers and 25.1% recent smokers (Table 1). Baseline recent smokers reported a longer lifetime smoking duration (median 29.0, IQR [24.0, 35.0] years) and higher cumulative pack-years (19.8 [11.4, 29.8]) than former smokers (14.0 [8.0, 22.0] years, and 7.8 [2.8, 16.2] pack-years, respectively). The median duration since quitting for ex-smokers at baseline was 16.0 years (IQR: [9.5, 23.5]). Amongst baseline ever-smokers (i.e., recent plus former; N = 14,367), 28.4% of the cohort participants (1232 females, 2844 males) met the German LCSC while 71.6% did not.

**Table 1:** Characteristics of participants from baseline to follow-up questionnaires

		Follow-up Round					
Variables		Baseline	2	3	4	5	6
**Participants (N (%))**		24715(100.0)	22410(90.7)	21597(87.4)	20688(83.7)	19680(79.6)	18405(74.5)
**Sex (N (%))**	*Men*	11594 (46.9; 46.9^**a**^)	10325 (41.8; 46.1)	9867(39.9; 45.7)	9382 (38.0; 45.3)	8854 (35.8; 45.0)	8189 (33.1; 44.5)
	*Women*	13121 (53.1; 53.1)	12085 (48.9; 53.9)	11730(47.5;54.3)	11306(45.7;54.7)	10826(43.8;55.0)	10216(41.3;55.5)
**Age (median, IQR; years)**		51.0(44.0, 58.0)	57.0(49.0, 63.0)	60.0(52.0, 66.0)	62.0(55.0, 69.0)	65.0(58.0, 71.0)	67.0(60.0, 74.0)
**Smoking status (N (%))**	*Never*	10348 (41.9; 41.9)	9426 (38.1; 42.1)	9098 (36.8; 42.1)	8749 (35.4; 42.3)	8361 (33.8; 42.5)	7865 (31.8; 42.7)
	*Former*	8160 (33.0; 33.0)	8220 (33.3; 36.7)	8082 (32.7; 37.4)	8608 (34.8; 41.6)	8517 (34.5; 43.3)	8188 (33.1; 44.5)
	*Recent*	6207 (25.1; 25.1)	4764 (19.3; 21.3)	4417 (17.9; 20.5)	3331 (13.5; 16.1)	2802 (11.3; 14.2)	2352 (9.5; 12.8)
**Duration of smoking (cumul.; years)** **(median; IQR)**	*Former*	14.0(8.0, 22.0)	16.0(9.0, 25.0)	16.0(9.0, 26.0)	17.0(9.0, 28.0)	18.0(10.0, 29.0)	18.0(10.0, 29.5)
	*Recent*	29.0(24.0, 35.0)	32.5(27.0, 39.0)	34.5(28.5, 41.0)	37.5(32.0, 43.5)	40.0(34.5, 46.0)	42.5(36.5, 48.0)
**Duration since quitting (cumul.; years)** **(median; IQR)**	*Former*	16.0(9.5, 23.5)	19.5(11.5, 28.0)	21.5(13.0, 30.5)	23.5(13.5, 32.5)	26.0(15.0, 35.0)	28.0(16.5, 37.5)
	*Recent*	0.0(0.0, 0.0)	0.0(0.0, 0.0)	0.0(0.0, 1.0)	0.0(0.0, 1.0)	0.0(0.0, 1.5)	0.0(0.0, 2.0)
**Pack years (cumul.)** **(median; IQR)**	*Former*	7.8(2.8, 16.2)	8.4(3.0, 18.0)	8.5(3.0, 18.1)	9.0(3.2, 19.2)	9.2(3.3, 19.7)	9.5(3.3, 20.0)
	*Recent*	19.8(11.4, 29.8)	22.1(12.6, 33.4)	23.2(13.0, 34.9)	26.0(15.3, 37.6)	27.5(16.2, 39.2)	28.3(16.5, 40.4)
**Lung cancer screening criteria** **for smokers (N (%))**	*met*	4076 (16.5; 28.4 ^**b**^)	3907 (15.8; 30.1)	3682 (14.9; 29.5)	3371 (13.6; 28.2)	2900 (11.7; 25.6)	2494 (10.1; 23.7)
	*not met*	10291 (41.6; 71.6)	9077 (36.7; 69.9)	8817 (35.7; 70.5)	8568 (34.7; 71.8)	8419 (34.1; 74.4)	8046 (32.6; 76.3)
**Deaths**^**c**^**(N (%))**		565(2.3; 24.5^**d**^)	819(3.3; 26.3)	1109(4.5; 27.5)	1491(6.0; 29.6)	1914(7.7; 30.3)	2974(12.0)
*Deaths from lung cancer (N (%))*		47(0.2; 2.0)	74(0.3; 2.4)	97(0.4; 2.4)	145(0.6; 2.9)	180(0.7; 2.9)	273(1.1)
*Deaths from other causes (N (%))*		518(2.1; 22.5)	745(3.0; 23.9)	1012(4.1; 25.1)	1346(5.4; 26.7)	1734(7.0; 27.5)	2701(10.9)
**Lost to follow up (N (%))**		1740(7.0; 75.5)	2299(9.3; 73.7)	2918(11.8; 72.5)	3544(14.3; 70.4)	4396(17.8; 69.7)	

^a^Two percentages are computed for discrete variables. The denominator for the first percentage is the total number of participants (24,715), while the denominator for the second percentage is the number of individuals who actually participated at the follow-up round

^b^The denominator for the second percentage is the number of ever smokers (include former smoker and recent smoker) who actually participated the follow-up round

^c^The right censoring time was set to the expected date of the next follow-up visit — 6 years after baseline and 3 years after the second to fifth visits. For participants who completed the last (sixth) follow-up round, the censoring time was defined as 5 years after that visit

Participants who died before the censoring time were considered to have experienced the event, whereas those who were permanently lost to follow-up were right-censored

^d^The denominator of the second percentage is the total number of participants who either died (event) or were censored due to loss to follow-up up to the expected censoring time

Over successive follow-up rounds, the numbers of participants reporting updates on their smoking behavior gradually decreased to 18,405 (8189 men, 10,216 women), and up to the 6th follow-up round a total of 6310 participants dropped out. The proportion of former smokers increased (from 33.0 to 44.5%) while the proportion of recent smokers decreased (25.1 to 12.8%) and the proportion of never smokers remained relatively stable (from 41.9 to 42.7%). From baseline till the sixth follow-up, the median cumulative smoking duration for recent smokers increased from 29.0 years (IQR: 24.0–35.0) to 42.5 years (IQR: 36.5–48.0), whereas for former smokers it increased from 14.0 (IQR: 8.0–22.0) to 18.0 (IQR: 10.0–29.5) years. Over time, the cumulative quitting duration for participants classified as former smokers progressively increased from baseline (median and IQR: 16.0 [9.5, 23.5]) to the sixth follow-up (28.0 [16.5, 37.5]). Amongst ever-smokers, the proportion that met German eligibility criteria for LC screening progressively decreased from 28.4 to 23.7%, due to increasing smoking cessation as participants grew older. A total of 2974 deaths were observed till the last censoring, of which 273 were due to LC and 2701 to other causes (Table 1).

Stratified by sex, analyses showed that men were less likely than women to complete follow-up until the 6th round (70.6% for men; 77.9% for women) (Additional file 1: Tables S1). Men had a higher baseline age (median [IQR]: 53.0 [46.6–58.4]) than women (49.0 [41.9–57.0]), and included a markedly lower proportion of never smokers (at baseline: 32.2% for men, 50.4% for women). Throughout the entire follow-up period, both former and recent male smokers had a longer cumulative smoking duration and higher cumulative pack-years, but for ex-smokers also longer duration since quitting, compared to the female participants. A greater proportion of men also met the LC screening criteria than women (e.g., at baseline: 24.5% for men, 9.4% for women). During follow-up, men had a higher mortality rate (16.8%) than women (7.8%), including both deaths from LC (1.7% for men; 0.6% for women) or from other causes (15.1% for men; 7.3% for women).

### Smoking-related variables (Continuous) and mortality risk

For both sexes, longer lifetime smoking duration and higher smoking intensity (cigarettes/day) were both significantly associated with higher risk of death either by LC or by other (non-LC) causes, as well as with all-cause mortality (Table 2). Among men, every additional 5 years of smoking duration was associated with a 35% increase in risk of death by LC (HR = 1.35 [95% CI: 1.27–1.44]) and a 7% increase in risk of death by other causes (HR = 1.07 [1.06–1.09]), whereas in women these increases were by about 19% (HR = 1.19 [1.11–1.27]) and 6% (HR = 1.06 [1.04–1.08]), respectively. Regarding intensity, every 10 cigarettes/day increase in smoking led to a 32% increase in LC-related death or a 23% increase in death by other causes in men, and a 70% increase in risk of LC death or 19% increase in mortality by other causes in women. With regard to smoking cessation, adjusting for lifetime smoking duration and intensity, longer duration since quitting were associated with moderate decreases in mortality by causes other than LC (for each addition 5 years of quitting, HR = 0.98 [95% CI: 0.97–1.00] in men, and HR = 0.97 [95% CI: 0.94–0.99] in women) as well as all-cause mortality, but not with LC-related mortality.

**Table 2:** Hazard ratios and P values for mortality by smoking-related continuous variables

	Men (n = 11594)		Women (n = 13121)	
	Hazard ratio (95% CI)	P value	Hazard ratio (95% CI)	P value
**Lung cancer mortality**	(event number = 198)		(event number = 75)	
Duration of smoking (per 5 yrs.)	1.35 (1.27–1.44)	<0.001	1.19 (1.11–1.27)	<0.001
Duration since quitting (per 5 yrs.)	1.01 (0.93–1.08)	0.88	0.97 (0.87–1.07)	0.52
Daily Cigarette Consumption	1.32 (1.17–1.48)	<0.001	1.70 (1.37–2.11)	<0.001
(per 10 Cigarettes/Day)				
**Other-cause mortality**	(event number = 1748)		(event number = 953)	
Duration of smoking (per 5 yrs.)	1.07 (1.06–1.09)	<0.001	1.06 (1.04–1.08)	<0.001
Duration since quitting (per 5 yrs.)	0.98 (0.97–1.00)	0.054	0.97 (0.94–0.99)	0.01
Daily Cigarette Consumption	1.23 (1.17–1.29)	<0.001	1.19 (1.05–1.35)	<0.001
(per 10 Cigarettes/Day)				
**All-cause mortality**	(event number = 1946)		(event number = 1028)	
Duration of smoking (per 5 yrs.)	1.09 (1.08–1.11)	<0.001	1.07 (1.04–1.09)	<0.001
Duration since quitting (per 5 yrs.)	0.98 (0.97–0.99)	0.01	0.96 (0.94–0.99)	0.01
Daily Cigarette Consumption	1.25 (1.19–1.31)	<0.001	1.26 (1.13–1.41)	<0.001
(per 10 Cigarettes/Day)				

- The right censoring time was set to the expected date of the next follow-up visit — 6 years after baseline and 3 years after the second to fifth visits. For participants who completed the last (sixth) follow-up round, the censoring time was defined as 5 years after that visit

Participants who died before the censoring time were considered to have experienced the event, whereas those who were permanently lost to follow-up were right-censored

- Time-varying Cox regression models were employed to evaluate the association of smoking-related continuous variables with the risk of mortality, using age as underlying time scale and with mutual adjustment between the smoking-related variables (but no further adjustments)

When considering LC incidence as the event, the HR associated with cumulative smoking duration was comparable to that observed for LC mortality in both sexes. Also in this analysis, longer cessation times (among former smokers) were not associated with a relative reduction in LC incidence, in either men or women, after adjustment for lifetime (past) smoking duration (Additional file 1: Tables S2).

### Mortality risk by age at quitting

**Fig. 1:**
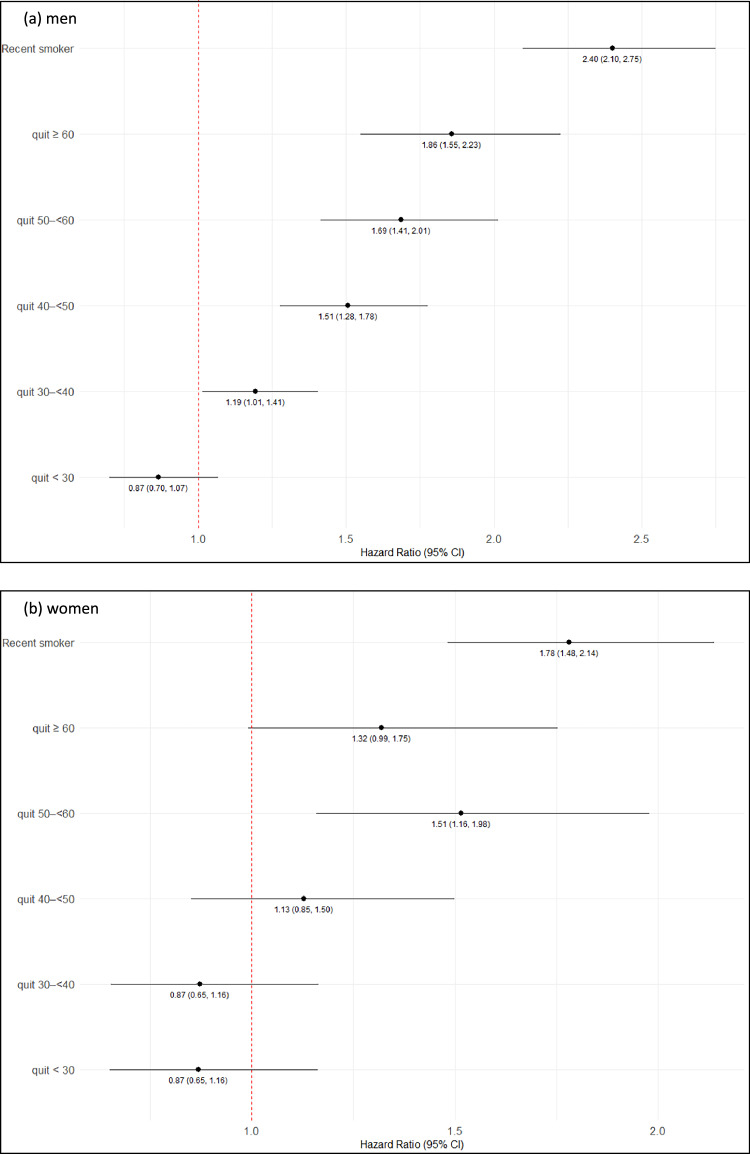
Hazard ratios for other-cause mortality for men (**a**) and women (**b**) by by age-at-quitting categories. Hazard ratios were estimated relative to never smokers as reference category, using a model with age as underlying time scale and without any further adjustments

Relative to never smokers, there was no significant increase in risk of other-cause mortality (Fig. 1) for men who quit smoking before the age of 30, or for women who quit before age 40. However, with increasing age at smoking cessation, the HRs gradually rose to 1.86 [1.55–2.23] for men who quit after age 60, and 1.51 [1.16–1.98] for women who quit at age 50–60 years. By comparison, the overall HRs for recent smoking (any age) were 2.40 [2.10–2.75] in men and 1.78 [1.48–2.14] in women. For all-cause mortality, the same relative risk patterns were observed in both men and women, but with slightly higher HR estimates (Additional file 1: Fig. S1).

### Smoking status and mortality risk, by age window

In analyses stratified by age window, depending on the age window and sex, recent smoking was associated with a 2 to 2.5-fold increase in other-cause mortality relative to never smokers (Table 3). In men, the HR for other-cause mortality among recent smokers went up from 2.07 for those below the age of 50 to 2.56 in the 60-<70 age range, followed by a moderate decline (to 2.55 [2.03–3.23]) for men aged 70 years and older. A similar pattern of increasing, and then decreasing HRs by age window, although of moderately lower magnitude, was observed for former smokers who had quit 2-<15 years, and ≥15 years ago. For women, other-cause mortality HRs for recent smokers ranged from 1.54 [1.05–2.25] in the 50-<60 age window to 2.43 [1.78–3.32] at ages above 70 years. In men, corresponding absolute incidence rates for other-cause mortality ranged from 1.7/1000 person years below age 50 to 17.2/1000 at age ≥70 for never smokers, and from 3.5/1000 to 36.2/1000, respectively, among recent smokers. In women, the incidence rates ranged from 0.9/1000 to 11.9/1000 in never smokers, and from 1.5/1000 to 23.8/1000 among recent smokers. As for lung cancer mortality, the HR among male recent smokers reached 54.40 (13.23–223.73) in the 60–<70 age range, followed by a marked decline to 13.03 (5.32–31.85) among those aged 70 years and older. When male smokers had quit smoking for ≥15 years, the HRs declined to approximately 3- to 7-fold compared with never smokers. A similar decreasing pattern across age windows was observed among women, though with considerably lower HRs. For women who had quit smoking ≥15 years prior, HRs were not significantly different from those of never smokers.

**Table 3:** Hazard ratios and absolute incidence rates of mortality by smoking status, sex, age window

**Men:** HRs (95% CI)			**<50**	**50-<60**	**60-<70**	**≥70**
*Incidence rates (Nr. of cases / total person years)*			*2.4 (78/32065)*	*4.6 (314/68071)*	*10.1 (727/72042)*	*23.4 (827/35268)*
**Never smoker**	*Lung cancer mortality*	HR	(ref.)	(ref.)	(ref.)	(ref.)
		*IR*	0 (0/9788)	0 (0/21617)	0.1 (2/24335)	0.5 (7/13320)
	*Other-cause mortality*	HR	(ref.)	(ref.)	(ref.)	(ref.)
		*IR*	*1.7 (17/9788)*	*2.8 (61/21617)*	*5.9 (143/24335)*	*17.2 (229/13320)*
	*All-cause mortality*	HR	(ref.)	(ref.)	(ref.)	(ref.)
		*IR*	*1.7 (17/9788)*	*2.8 (61/21617)*	*6.0 (145/24335)*	*17.7 (236/13320)*
**Former smoker**						
quit ≥15 years	*Lung cancer mortality*	HR	NA	NA	7.97 (1.85–34.34)	3.12 (1.36–7.18)
		*IR*	*0 (0/5338)*	*0.1 (2/19502)*	*0.7 (18/27131)*	*1.6 (26/15867)*
	*Other-cause mortality*	HR	1.13 (0.53–2.41)	1.24 (0.88–1.75)	1.25 (1.01–1.55)	1.23 (1.04–1.46)
		*IR*	*2.1 (11/5338)*	*3.5 (69/19502)*	*7.5 (202/27131)*	*21.2 (336/15867)*
	*All-cause mortality*	HR	1.14 (0.53–2.42)	1.28 (0.91–1.80)	1.35 (1.09–1.66)	1.29 (1.09–1.52)
		*IR*	*2.1 (11/5338)*	*3.6 (71/19502)*	*8.1 (220/27131)*	*22.8 (362/15867)*
quit 2-<15 years	*Lung cancer mortality*	HR	NA	NA	38.55 (9.18–161.84)	10.94 (4.49–26.66)
		*IR*	*0 (0/6201)*	*0.5 (5/10431)*	*3.1 (28/9132)*	*5.3 (17/3209)*
	*Other-cause mortality*	HR	0.84 (0.37–1.89)	1.60 (1.09–2.35)	2.33 (1.83–2.97)	1.86 (1.45–2.37)
		*IR*	*1.5 (9/6201)*	*4.4 (46/10431)*	*13.3 (122/9132)*	*28.4 (91/3209)*
	*All-cause mortality*	HR	0.84 (0.37–1.89)	1.77 (1.22–2.57)	2.83 (2.25–3.56)	2.14 (1.70–2.69)
		*IR*	*1.5 (9/6201)*	*4.9 (51/10431)*	*16.4 (150/9132)*	*33.7 (108/3209)*
**Recent smoker**	*Lung cancer mortality*	HR	NA	NA	54.40 (13.23–223.73)	13.03 (5.32–31.85)
		*IR*	*0.3 (3/10738)*	*1.5 (25/16521)*	*4.2 (48/11444)*	*5.9 (17/2872)*
	*Other-cause mortality*	HR	2.07 (1.17–3.67)	2.38 (1.72–3.23)	2.56 (2.04–3.20)	2.55 (2.03–3.23)
		*IR*	*3.5 (38/10738)*	*6.4 (106/16521)*	*14.3 (164/11444)*	*36.2 (104/2872)*
	*All-cause mortality*	HR	2.23 (1.27–3.93)	2.91 (2.15–3.94)	3.27 (2.65–4.04)	2.88 (2.31–3.59)
		*IR*	*[3.8 (41/10738)]*	*[7.9 (131/16521)]*	*[18.5 (212/11444)]*	*[42.1 (121/2872)]*
**Women:** **HRs (95%CI)**			**<50**	**50-**<**60**	**60-**<**70**	**≥70**
*Incidence rates (Nr. of cases/total person years)*			*1.2 (72/62714)*	*2.2 (179/81986)*	*4.5 (312/68985)*	*24.7 (827/33461)*
**Never smoker**	*Lung cancer mortality*	HR	(ref.)	(ref.)	(ref.)	(ref.)
		*IR*	*0 (0/25300)*	0 (1/37382)	0.2 (8/38689)	0.4 (10/22348)
	*Other-cause mortality*	HR	(ref.)	(ref.)	(ref.)	(ref.)
		*IR*	*0.9 (23/25300)*	*1.9 (70/37382)*	*3.9 (151/38689)*	*11.9 (265/22348)*
	*All-cause mortality*	HR	(ref.)	(ref.)	(ref.)	(ref.)
		*IR*	*0.9 (23/25300)*	*1.9 (71/37382)*	*4.1 (159/38689)*	*12.3 (275/22348)*
**Former smoker**						
quit ≥15 years	*Lung cancer mortality*	HR	NA	5.89 (0.60–57.81)	1.21 (0.37–4.02)	1.97 (0.72–5.38)
		*IR*	*0 (0/9566)*	*0.2 (3/19928)*	*0.3 (4/16227)*	*0.8 (6/7152)*
	*Other-cause mortality*	HR	0.75 (0.32–1.77)	1.04 (0.70–1.55)	0.79 (0.58–1.09)	1.14 (0.90–1.45)
		*IR*	*0.7 (7/9566)*	*1.8 (37/19928)*	*3.0 (50/16227)*	*13.0 (93/7152)*
	*All-cause mortality*	HR	0.75 (0.32–1.77)	1.11 (0.75–1.64)	0.81 (0.60–1.11)	1.17 (0.93–1.47)
		*IR*	*0.7 (7/9566)*	*2.0 (40/19928)*	*3.3 (54/16227)*	*13.8 (99/7152)*
quit 2-<15 years	*Lung cancer mortality*	HR	NA	4.86 (0.30–79.23)	3.34 (1.01–11.07)	5.26 (1.67–16.57)
		*IR*	*0.1 (1/10255)*	*0.1 (1/8252)*	*0.7 (4/5812)*	*2.1 (4/1906)*
	*Other-cause mortality*	HR	1.35 (0.67–2.69)	1.11 (0.64–1.92)	1.21 (0.81–1.83)	1.77 (1.23–2.54)
		*IR*	*1.2 (12/10255)*	*1.9 (16/8252)*	*4.7 (27/5812)*	*17.8 (34/1906)*
	*All-cause mortality*	HR	1.46 (0.74–2.86)	1.17 (0.68–1.99)	1.32 (0.90–1.94)	1.90 (1.35–2.68)
		*IR*	*1.3 (13/10255)*	*2.1 (17/8252)*	*5.3 (31/5812)*	*19.9 (38/1906)*
**Recent smoker**	*Lung cancer mortality*	HR	NA	16.98 (2.02–142.92)	11.17 (4.87–25.65)	4.98 (1.58–15.70)
		*IR*	*0.2 (3/17593)*	*0.4 (7/16424)*	*2.3 (19/8257)*	*2.0 (4/2055)*
	*Other-cause mortality*	HR	1.66 (0.95–2.91)	1.54 (1.05–2.25)	1.60 (1.16–2.21)	2.43 (1.78–3.32)
		*IR*	*1.5 (26/17593)*	*2.7 (44/16424)*	*5.9 (49/8257)*	*23.8 (49/2055)*
	*All-cause mortality*	HR	1.85 (1.07–3.20)	1.75 (1.22–2.53)	2.10 (1.58–2.79)	2.53 (1.88–3.41)]
		*IR*	*1.7 (29/17593)*	*3.1 (51/16424)*	*8.2 (68/8257)*	*25.8 (53/2055)*

- To estimate age-specific relative risks of mortality, participants were classified into the corresponding age groups according to their age at each follow-up. Thus, the same participant could contribute to different age groups as their age increased across multiple follow-ups. Hazard ratios and their 95% confident interval for each age group were then estimated using time-dependent Cox regression models, with age as underlying time scale and without further adjustments

- The unit of the incidence rate is per 1000 person-years

- Time-varying Cox regression models were employed to evaluate the association of smoking status with the risk of mortality, using age as underlying time scale and without further adjustments

For all-cause mortality, HRs and absolute incidence rates by smoking status, sex and age window were slightly higher than those for other-cause (non-LC) mortality (Table 3).

### Lung cancer screening criteria and mortality risk, by age window

Considering smoking men who met the eligibility status according to the LCSC (“current eligible smokers”) (Table 4), approximately 2.4 to 2.7-fold HRs for other-cause mortality were estimated within the 50-<60, 60-<70, 70-<75, and 75–80- year age windows. For men who were once eligible, but had then quit smoking for more than 10 years (“past eligible” smokers), average quitting times increased from 13 years in the 50-<60- year age window to 22 years when participants were 75-<80 years old, and the HRs for other-cause mortality fell from about 3-fold in the 50-<60-year age window to about 1.5-fold in the 75-<80- year age window. For smokers who never became eligible for screening, relative risk estimates for other-cause mortality were close to unity (“never-eligible smokers”). In women, HRs for other-cause mortality in screening-eligible smokers vs never-smokers increased from about 2-fold in the 50-<60- year to the age window to about 3-fold above the age of 70. For smokers who never reached eligibility for screening, no increase in the relative risks for other-cause mortality was observed.

**Table 4:** Hazard ratios and absolute incidence rates of mortality by LC screening criteria, sex, age window

**Men:** HRs (95% CI)			**50-<60**	**60-<70**	**70-<75**	**75-<80**
[Incidence rates (Nr. of cases / total person years)]			*4.6 (314/68072)*	*10.1 (727/72042)*	*16.7 (377/22541)*	*32.0 (351/10962)*
**Never smoker**	*Lung cancer mortality*	HR	(ref.)	(ref.)	(ref.)	(ref.)
		*IR*	*0 (0/21617)*	*0.1 (2/24335)*	*0.6 (5/8206)*	*0.4 (2/4425)*
	*Other-cause mortality*	HR	(ref.)	(ref.)	(ref.)	(ref.)
		*IR*	*2.8 (61/21617)*	*5.9 (143/24335)*	*11.8 (97/8206)*	*22.4 (99/4425)*
	*All-cause mortality*	HR	(ref.)	(ref.)	(ref.)	(ref.)
		*IR*	*2.8 (61/21617)*	*6.0 (145/24335)*	*12.4 (102/8206)*	*20.9 (101/4425)*
**Never-eligible Smokers**	*Lifetime smoking /quitting duration*	median	15 / 21.5	15 / 30	15 /36.5	16 / 41
	*Lung cancer mortality*	HR	NA	4.04 (0.87–18.72)	0.39 (0.07–2.00)	3.28 (0.67–16.17)
		*IR*	*0.1 (2/26922)*	*0.3 (9/27337)*	*0.2 (2/8505)*	*1.5 (6/4003)*
	*Other-cause mortality*	HR	1.11 (0.80–1.55)	1.20 (0.97–1.49)	(0.77–1.34)	1.33 (1.02–1.73)
		*IR*	*3.1 (83/26922)*	*7.0 (192/27337)*	*12.0 (102/8505)*	*29.5 (118/4003)*
	*All-cause mortality*	HR	1.13 (0.82–1.58)	1.24 (1.00–1.54)	0.98 (0.75–1.29)	1.36 (1.05–1.77)
		*IR*	*3.2 (85/26922)*	*7.4 (201/27337)*	*12.2 (104/8505)*	*31.0 (124/4003)*
**Past-eligible Smokers**	*Lifetime smoking /quitting duration*	median	27 / 13	30 / 16	33 / 19.5	35 / 22
	*Lung cancer mortality*	HR	NA	33.98 (7.91–145.91)	3.33 (1.02–10.88)	15.77 (3.53–70.54)
		*IR*	*1.5 (3/2057)*	*2.9 (19/6486)*	*2.0 (6/2944)*	*7.1 (11/1536)*
	*Other-cause mortality*	HR	2.99 (1.79–5.01)	1.95 (1.48–2.57)	1.78 (1.30–2.45)	1.53 (1.09–2.14)
		*IR*	*9.2 (19/2057)*	*11.9 (77/6486)*	*21.1 (62/2944)*	*33.9 (52/1536)*
	*All-cause mortality*	HR	3.49 (2.15–5.69)	2.39 (1.85–3.10)	1.86 (1.37–2.53)	1.81 (1.32–2.49)
		*IR*	*10.7 (22/2057)*	*14.8 (96/6486)*	*23.1 (68/2944)*	*41.0 (63/1536)*
**Current-eligible Smokers**	*Lifetime smoking /quitting duration*	median	36 / 0	43.5 / 0	50 / 1	53.5 / 2
	*Lung cancer mortality*	HR	NA	60.71(14.87–247.81)	13.34 (5.06–35.16)	11.05 (2.15–56.73)
		*IR*	*1.5 (27/17476)*	*4.7 (66/13883)*	*23/2888 (8.0)*	*5/998 (5.0)*
	*Other-cause mortality*	HR	2.46 (1.81–3.35)	2.79 (2.26–3.44)	2.40 (1.79–3.22)	2.73 (1.97–3.78)
		*IR*	*6.8 (119/17476)*	*15.8 (219/13883)*	*27.7 (80/2888)*	*58.1 (58/998)*
	*All-cause mortality*	HR	3.01 (2.24–4.06)	3.58 (2.93–4.38)	2.94 (2.24–3.85)	2.89 (2.11–3.97)
		*IR*	*8.4 (146/17476)*	*20.5 (285/13883)*	*35.7 (103/2888)*	*63.1 (63/998)*
**Women:** **HRs (95%CI)**			**50-**<**60**	**60-**<**70**	**70-**<**75**	**75-**<**80**
[Incidence rates (Nr. of cases / total person years)]			*2.2 (179/81986)*	*4.5 (312/68986)*	*9.6 (201/20949)*	*18.6 (197/10576)*
**Never smoker (ref.)**	*Lung cancer mortality*	HR	(ref.)	(ref.)	(ref.)	(ref.)
		*IR*	*0 (1/37382)*	*0.2 (8/38689)*	*0.3 (4/13509)*	*0.7 (5/7457)*
	*Other-cause mortality*	HR	(ref.)	(ref.)	(ref.)	(ref.)
		*IR*	*1.9 (70/37382)*	*3.9 (151/38689)*	*7.6 (103/13509)*	*15.4 (115/7457)*
	*All-cause mortality*	HR	(ref.)	(ref.)	(ref.)	(ref.)
		*IR*	*1.9 (71/37382)*	*4.1 (159/38689)*	*7.9 (107/13509)*	*16.1 (120/7457)*
**Never-eligible Smokers**	*Lifetime smoking /quitting duration*	median	15 / 21	15.5 / 27	16 / 31.5	17.5 / 34.5
	*Lung cancer mortality*	HR	3.79 (3.80–3.78)	2.35 (9.28–5.93)	2.07 (0.47–9.20)	1.42 (0.28–7.26)
		*IR*	*0.1 (3/31444)*	*0.5 (10/20779)*	*0.6 (3/5083)*	*0.9 (2/2148)*
	*Other-cause mortality*	HR	1.00 (0.70–1.43)	0.78 (0.58–1.05)	1.02 (0.71–1.47)	1.34 (0.94–1.90)
		*IR*	*1.7 (55/31444)*	*3.0 (62/20779)*	*7.7 (39/5083)*	*20 (43/2148)*
	*All-cause mortality*	HR	1.04 (0.73–1.48)	0.86 (0.65–1.14)	1.06 (0.74–1.51)	1.34 (0.95–1.89)
		*IR*	*1.8 (58/31444)*	*3.5 (72/20779)*	*8.3 (42/5083)*	*20.9 (45/2148)*
**Past-eligible Smokers**	*Lifetime smoking /quitting duration*	median	27 / 12.5	31 / 15	34 / 19.5	37 / 19
	*Lung cancer mortality*	HR	NA	NA	7.93 (1.48, 42.63)	NA
		*IR*	*0 (0/731)*	*0 (0/1903)*	*2.4 (2/840)*	*0 (0/437)*
	*Other-cause mortality*	HR	0.68 (0.09–4.89)	1.35 (0.73–2.49)	1.87 (1.04–3.36)	1.50 (0.78–2.88)
		*IR*	*1.4 (1/731)*	*5.8 (11/1903)*	*14.3 (12/840)*	*22.9 (10/437)*
	*All-cause mortality*	HR	0.67 (0.09–4.82)	1.30 (0.70–2.39)	2.10 (1.22–3.61)	1.43 (0.74–2.75)
		*IR*	*1.4 (1/731)*	*5.8 (11/1903)*	*16.7 (14/840)*	*22.9 (10/437)*
**Current-eligible Smokers**	*Lifetime smoking /quitting duration*	median	36 / 0	43.5 / 0	49 / 0	53.5 / 1
	*Lung cancer mortality*	HR	25.14 (3.08–205.31)	10.86 (4.68–25.21)	9.20 (2.30–36.71)	6.38 (1.26–32.20)
		*IR*	*0.6 (8/12430)*	*2.2 (17/7615)*	*2.6 (4/1517)*	*3.7 (2/534)*
	*Other-cause mortality*	HR	1.86 (1.26–2.74)	1.85 (1.35–2.52)	2.99 (2.03–4.41)	2.67 (1.65–4.32)
		*IR*	*3.3 (41/12430)*	*7.0 (53/7615)*	*22.4 (34/1517)*	*37.5 (20/534)*
	*All-cause mortality*	HR	2.19 (1.52–3.16)	2.31 (1.74–3.06)	3.22 (2.22–4.67)	2.82 (1.78–4.48)
		*IR*	*3.9 (49/12430)*	*9.2 (70/7615)*	*25.0 (38/1517)*	*41.2 (22/534)*

- To estimate age-specific relative risks of mortality, participants were classified into the corresponding age groups according to their age at each follow-up. Thus, the same participant could contribute to different age groups as their age increased across multiple follow-ups. Hazard ratios and their 95% confident interval for each age group were then estimated using time-dependent Cox regression models, with age as underlying time scale and without further adjustments

- Based on the German lung cancer screening criteria, this study applied left censoring by excluding information from follow-up visits before age 50, and right censoring by extending the upper age limit to 80 years

- The unit of the incidence rate is per 1000 person-years

In never-smoking men, absolute incidence rates for other-cause mortality ranged from 2.8/1000 person years at age 50-<60 to 11.8/1000 at age 70-<75 and 22.4/1000 at age 75-<80, compared to 6.8/1000 (age 50-<60) to 27.7/1000 (age 70-<75) or 58.1/1000 (age 75-<80) among current eligible smokers, and 9.2/1000 (age 50-<60) to 33.9/1000 (age 75-<80) in men who were once eligible, but then lost eligibility because of more than 10 years’ smoking cessation (Table 4). Of note, the incidence rate for other-cause mortality amongst past-eligible smokers age 75-<80 (33.9/1000) appeared to be not much higher than that among eligible smokers of the younger, 70-<75-year age group (27.7/1000).

In women, absolute rates for other-cause mortality showed an overall pattern by age and eligibility status similar to that observed in men, although absolute rates were lower.

For all-cause mortality, the patterns of relative risk and absolute incidence rates were very similar to those for other-cause mortality. For LC, relative mortality hazards were substantially higher than those for mortality by other causes.

## Discussion

We evaluated the association between smoking history and mortality risk based on the EPIC-Heidelberg cohort, using repeatedly measured smoking information over a period of up to 20 years. Relative to never-smokers, recent smoking significantly increased the relative risks of dying from LC as well as from other causes, and resulted in approximately 2- to 3-fold increased risks of all-cause mortality in, respectively, women or men above the age of 60. Compared to never-smokers, smokers who met the German LCSC also had up to 3-fold increased risk death by causes other than LC as well as for all-cause mortality. Among former smokers, long-term quitters (>15 years), and especially those who quit at a young age (<35 years), showed no or only weakly increased risks of death by causes other than LC, relative to never smokers. However, for those who had smoked long enough to meet the official LCSC, the relative risks for other-cause and overall mortality remained elevated compared to never smokers even after 10 years of quitting, although less strongly so than those who continued smoking.

Our descriptive analyses showed that between 1994 and 2014 the overall proportion of former smokers progressively increased as participants grew older, in line with cross-sectional findings from German population surveys, which also document an increasing prevalence of former smokers for older, as compared to younger age groups [25]. Furthermore, our findings from the EPIC-Heidelberg cohort showed a higher overall prevalence of recent smoking, as well as intensity and lifetime duration, among men relative to women, matched by higher mortality rates both for LC and other causes. Besides age, German population surveys and our data from the EPIC-cohort show that smoking behaviors also differed across birth cohorts, and that especially among women smoking prevalence had increased for post-War birth cohorts. Finally, smoking status changed longitudinally with age in both men and women, and with advancing age, an increasing number of smokers quit or progressively smoked fewer cigarettes. Thus, the combined effects of sex, age, and birth cohort on smoking behavior in our study were generally consistent with findings from cross-sectional analyses in German population surveys [25–29], although within the same age windows and calendar years, both men and women in the EPIC-Heidelberg cohort showed lower absolute smoking prevalence than the average German population [25,26,28]. The somewhat lower absolute smoking prevalence in the EPIC-Heidelberg cohort, relative to the average German population, may be due to differences in educational attainment and occupational status [26,27,29], as Heidelberg and its surrounding regions, being a university city, are characterized by generally higher levels of education and occupational status.

For recent smokers, our estimates for relative mortality risks among men and women are comparable to those from large-scale cohort studies conducted in the United States [5,7,10,22] or the United Kingdom [8]. These studies, which also collected data in the 1990s and 2000s, showed that men or women with a median or mean baseline age of about 50+ [5,7,8,22] or even 60+ years [10], who had a long history of smoking and had not quit for an extended period, had about 2 to 3-fold higher risks of all-cause mortality compared to never smokers over ten or more years of follow-up [5,7,8,10,21,22]. Regarding the effects of smoking by sex, however, some of the studies showed approximately equal increases in mortality for both women and men [5,7,10,22], whereas other studies [1,11] showed somewhat stronger relative risks for men than for women, similar to our findings.

Regarding smoking cessation, earlier cohort studies generally indicated that the relative risk of all-cause mortality declined with longer duration since cessation or earlier age at quitting, and that after more than 15–20 years of sustained abstinence the relative risk for all-cause mortality was no longer significantly different from that of never smokers [5,7,8,10,22], consistent with the results of our study. Similar to an analysis US National Health Interview Survey [22], our findings from the EPIC-Heidelberg cohort indicate that, relative to never smokers, the risk for all-cause mortality, as well as for mortality by causes other than LC, progressively increases with age at quitting, with no increase for men or women who quit before 30 and 40 respectively, but 118 and 42% increased all-cause mortality risks for those quitting over 60 years old.

Specifically for LC, lifetime smoking duration and smoking intensity were very strong determinants of both incidence and related mortality risk, with HRs that much exceeded those for mortality by other causes [5,7,8,10,22]. However, adjusting for lifetime former smoking duration, our data showed no major relative risk reduction for LC incidence or mortality in relation to time since quitting.

Since 2024, Germany authorizes LC screening by low-dose computed tomography (LDCT) based on the mentioned LCSC, i.e., in men or women age 50–75 who have smoked ≥25 years and with a cumulative smoking exposure of at least 15 pack-years, but who did not quit smoking more than 10 years ago [13] However, it is debated whether individuals who at some point have met these requirements, but then quit smoking for more than 10 years (“past-eligible smokers”), should no longer be eligible for screening [18,19]. The motivation for excluding quitters from further screening is the assumption that progressively the risk of having LC drops with smoking cessation time. However, epidemiologic studies (including ours) have shown that former smokers maintain an elevated risk for LC relative to never smokers, and also that absolute risk of developing LC does not decrease after quitting [1,17,30].

Besides the question how smoking cessation affects LC risk and mortality, an accompanying question is how it affects risk of death by other causes. A rational approach to determining when LDCT screening should be stopped would be based on an estimate of absolute risk for other-cause mortality, e.g. over the next five years. In fact, for individuals who once at some point meet the LCSC, but then quit smoking, the benefit-to-harm balance for LDCT screening likely could be better than that for individuals who keep smoking, as quitters may have a somewhat lower risk of death by causes other than LC, whereas the risk of having LC does not itself diminish. Overall, our analyses show that, while early-in-life quitters have little excess mortality risk, a significant increase in risk for other-cause (non-LC) mortality remains for smokers who quit after first having met German eligibility criteria for LC screening (i.e., individuals who smoked >15 pack-years and who had been smoking relatively recently up to the age of 50 or more years).

While most previous cohort studies on smoking and mortality risk were based on only a single baseline ascertainment of smoking history, without accounting for future changes in smoking habits during longer-term prospective follow-up, a strength of our study is that it made use of regular, repeat ascertainments of smoking status and intensity over an approximately 20-year period. We could thus estimate relative risks associated with recent smoking status or quitting times with greater accuracy, for mortality outcomes occurring over the next 3–5 years. A limitation of our study, however, is that it was considerably smaller than some of the previous cohort analyses and that it included only about 3000 incident cases of mortality. Further, larger-scale studies may be needed to obtain more precise estimates for other-cause mortality risks and residual life-expectancy for screening-eligible individuals who quit smoking, relative to those who continue, and to determine optimized maximum ages for LC screening in long-term recent *versus* former smokers.

## Conclusions

In summary, our findings confirm that, relative to never smokers, the risk of developing or dying from LC does not significantly diminish after smoking cessation, in line with findings from previous studies [1,17,30]. This observation argues against excluding individuals from LC screening after smoking cessation, if at a previous point in time they met eligibility criteria, and hence, had reached a minimally required absolute LC risk [17–19]. However, with regard to the reverse question, i.e., whether longer-term quitters who had once reached LCSC could not be granted a later stopping age, compared to screening participants who continued smoking, our findings are somewhat mitigated. Our estimates suggest that the risk of other-cause mortality remains elevated, but less so than for continuing smokers, with absolute incidence rates in past-eligible smokers in the age window of 75-<80 that were comparable to those for current-eligible smokers in the younger, 70-<75-year age window. These estimates might indeed argue for a moderate extension of the maximum age limit for screening, relative to continuing smokers, but larger studies will be needed to obtain more precise risk estimates.

## Supplementary information

Additional File 1 **Method S1** - Specific method to calculate and impute smoking-related variables. **Table S1** Characteristics of participants from baseline to follow-up questionnaires by sex in the EPIC-Heidelberg cohort. **Table S2** Hazard ratios and P values for lung cancer incidence risks by smoking-related continuous variables in the EPIC-Heidelberg cohort. **Figure S1** - Hazard ratios for all-cause mortality by sex and age-at-quitting categories in the EPIC-Heidelberg cohort

## Data Availability

EPIC-Heidelberg was launched in the 1990s. Unlike in new studies that we run today, public access to data from the EPIC population was not part of the study protocol at that time. Thus, the data protection statement and informed consent of the EPIC participants do not cover the provision of data in public repositories. Nevertheless, upon special request data may be made available for (a) statistical validation by reviewers and (b) pooling projects under clearly defined and secure conditions and based on valid data transfer agreements.

## Abbreviations

LC: lung cancer
LCSC: lung cancer screening criteria
LDCT: Low-Dose Computed Tomography
EPIC: European Prospective Investigation into Cancer and Nutrition
HR: hazard ratio
IQR: inter-quartile range
CI: confidence interval
