# Supplementary material for: Smoking history, eligibility for lung cancer screening and risk of death by lung cancer or other causes -- a longitudinal, time-variable analysis of the EPIC-Heidelberg cohort
Source: BMC Med. 2026 Apr 28;24:274. doi: 10.1186/s12916-026-04891-z (PMC13126791; doi:10.1186/s12916-026-04891-z)
Supplement: Supplementary file 1 — Additional File 1 Method S1 - Specific method to calculate and impute smoking-related variables. Table S1 Characteristics of participants from baseline to follow-up questionnaires by sex in the EPIC-Heidelberg cohort. Table S2 Hazard ratios and P values for lung cancer incidence risks by smoking-related continuous variables in the EPIC-Heidelberg cohort. Figure S1 - Hazard ratios for all-cause mortality by sex and age-at-quitting categories in the EPIC-Heidelberg cohort [file 12916_2026_4891_MOESM1_ESM.docx]

**Additional File 1**
**Method S1: Specific method to calculate and impute smoking-related variables**

To effectively handle missing values in smoking-related variables, we made the following assumptions:

1. We estimated smoking information only for participants who either completed all follow-up rounds or had intermittent missing visits but later re-engaged. No data were imputed for those permanently lost to follow-up after a given time point.
2. Smoking status at each follow-up was simplified into two categories: smoking and non-smoking.
3. If smoking status remained unchanged between two consecutive follow-ups, the participant was assumed to maintain that status throughout the interval, and the corresponding smoking or cessation duration was calculated accordingly.
4. When smoking status changed between two follow-ups, two scenarios were considered. For transitions from smoking to non-smoking, if the age at quitting was reported, smoking duration was calculated from the age of initiation to the age at quitting, and cessation duration from quitting to the next follow-up. If the age at quitting was missing, an equal split (half smoking, half cessation) was assumed. For transitions from non-smoking to smoking, as no detailed information on relapse or initiation age was collected in subsequent follow-up rounds, an equal split was also assumed for simplicity.
5. Estimating daily smoking consumption (cigarettes/day) was more challenging, as irregular changes may occur even when values at two consecutive follow-ups are available. Therefore, linear interpolation was applied to estimate missing consumption values between follow-ups.

After applying these rules, 1,713 smokers still had consistently missing information on daily cigarette consumption. As this variable is closely related to sex, age of initial smoking, cumulative smoking duration, and cumulative cessation duration, it was considered to satisfy the Missing at Random (MAR) assumption. Consequently, multiple imputation by chained equations (MICE) was used, assuming a normal distribution for daily cigarette consumption.

**Table S1: (A) Characteristics of participants from baseline to follow-up questionnaires by sex in the EPIC-Heidelberg cohort (men)**

|  | | **Follow-up Round** | | | | | |
| --- | --- | --- | --- | --- | --- | --- | --- |
| **Variables** | | **Baseline** | **2** | **3** | **4** | **5** | **6** |
| **Participants (N (%))** | | 11594 (100.0) | 10325 (89.1) | 9867 (85.1) | 9382 (80.9) | 8854 (76.4) | 8189 (70.6) |
| **Age (median, IQR; years)** | | 53.0(46.6, 58.4) | 58.6(52.2, 63.9) | 61.4(54.9, 66.7) | 64.2(57.8, 69.5) | 67.0(60.6, 72.3) | 69.2(62.8, 74.6) |
| **Smoking status (N (%))** | *Never* | 3739 (32.2; 32.2 **^a^**) | 3401 (29.3; 32.9) | 3264 (28.2; 33.1) | 3129 (27.0; 33.4) | 2987 (25.8; 33.7) | 2775 (23.9; 33.9) |
|  | *Former* | 4643 (40.0; 40.0) | 4594 (39.6; 44.5) | 4470 (38.6; 45.3) | 4704 (40.6; 50.1) | 4630 (39.9; 52.3) | 4389 (37.9; 53.6) |
|  | *Recent* | 3212 (27.7; 27.7) | 2330 (20.1; 22.6) | 2133 (18.4; 21.6) | 1549 (13.4; 16.5) | 1237 (10.7; 14.0) | 1025 (8.8; 12.5) |
| **Duration of smoking (cumul.; years)**  **(median; IQR)** | *Former* | 16.0(10.0, 24.0) | 18.0(10.0, 27.0) | 18.0(10.0, 28.0) | 19.0(11.0, 30.0) | 20.0(11.0, 30.5) | 20.0(11.0, 31.0) |
|  | *Recent* | 31.5(26.0, 37.5) | 35.0(30.0, 41.5) | 36.5(30.5, 43.5) | 40.0(34.5, 46.0) | 42.5(36.5, 48.5) | 44.5(38.5, 50.0) |
| **Duration since quitting (cumul.; years) (median; IQR)** | *Former* | 17.0(9.5, 24.5) | 21.0(11.5, 29.5) | 23.0(13.5, 32.0) | 24.5(14.0, 34.0) | 27.0(16.0, 36.5) | 29.0(17.5, 39.0) |
|  | *Recent* | 0.0(0.0, 0.0) | 0.0(0.0, 0.0) | 0.0(0.0, 1.0) | 0.0(0.0, 1.0) | 0.0(0.0, 1.5) | 0.0(0.0, 2.5) |
| **Pack years (cumul.)**  **(median; IQR)** | *Former* | 11.2(4.5, 21.0) | 12.1(5.0, 22.5) | 12.4(5.0, 23.0) | 13.0(5.0, 24.0) | 13.0(5.2, 24.7) | 13.3(5.2, 25.0) |
|  | *Recent* | 25.5(16.3, 35.4) | 28.8(17.8, 38.9) | 29.7(17.8, 40.9) | 32.5(20.7, 44.1) | 33.6(21.4, 46.2) | 34.6(21.1, 47.5) |
| **Lung cancer screening criteria**  **for smokers (N (%))** | *met* | 2844 (24.5; 36.2 **^b^**) | 2379 (20.5; 34.4) | 2127 (18.3; 32.2) | 1866 (16.1; 29.8) | 1523 (13.1; 26.0) | 1270 (11.0; 23.5) |
|  | *not met* | 5011 (43.2; 63.8) | 4545 (39.2; 65.6) | 4476 (38.6; 67.8) | 4387 (37.8; 70.2) | 4344 (37.5; 74.0) | 4144 (35.7; 76.5) |
| **Deaths ^c^ (N (%))** | | 394(3.4; 31.0 **^d^**) | 569(4.9; 32.9) | 752(6.5; 34.0) | 994(8.6; 36.3) | 1277(11.0; 37.5) | 1946(16.8) |
| *Deaths from lung cancer (N (%))* | | 38(0.3; 3.0) | 61(0.5; 3.5) | 77(0.7; 3.5) | 111(1.0; 4.1) | 132(1.1; 3.9) | 198(1.7) |
| *Deaths from other causes (N (%))* | | 356(3.1; 28.1) | 508(4.4; 29.4) | 675(5.8; 30.5) | 883(7.6; 32.2) | 1145(9.9; 33.6) | 1748(15.1) |
| **Lost to follow up (N (%))** | | 875(7.5; 69.0) | 1158(10.0; 67.1) | 1460(12.6; 66.0) | 1746(15.1; 63.7) | 2128(18.4; 62.5) |  |

1. Two percentages are computed for discrete variables. The denominator for the first percentage is the total number of male participants (11, 594), while the denominator for the second percentage is the number of male individuals who actually participated at the follow-up round.
2. The denominator for the second percentage is the number of ever male smokers (include former smoker and recent smoker) who actually participated the follow-up round.
3. The right censoring time was set to the expected date of the next follow-up visit — 6 years after baseline and 3 years after the second to fifth visits. For participants who completed the last (sixth) follow-up round, the censoring time was defined as 5 years after that visit.

Participants who died before the censoring time were considered to have experienced the event, whereas those who were permanently lost to follow-up were right-censored.

1. The denominator of the second percentage is the total number of male participants who either died (event) or were censored due to loss to follow-up up to the expected censoring time.

**Table S1: (B) Characteristics of participants from baseline to follow-up questionnaires by sex in the EPIC-Heidelberg cohort (women)**

|  | | **Follow-up Round** | | | | | |
| --- | --- | --- | --- | --- | --- | --- | --- |
| **Variables** | | **Baseline** | **2** | **3** | **4** | **5** | **6** |
| **Participants (N (%))** | | 13121 (100.0) | 12085 (92.1) | 11730 (89.4) | 11306 (86.2) | 10826 (82.5) | 10216 (77.9) |
| **Age (median, IQR; years)** | | 49.0(41.9, 57.0) | 54.6(47.5, 62.4) | 57.4(50.3, 65.0) | 60.2(53.2, 67.7) | 63.0(56.1, 70.4) | 65.3(58.5, 72.7) |
| **Smoking status (N (%))** | *Never* | 6609 (50.4; 50.4 **^a^**) | 6025 (45.9; 49.9) | 5834 (44.5; 49.7) | 5620 (42.8; 49.7) | 5374 (41.0; 49.6) | 5090 (38.8; 49.8) |
|  | *Former* | 3517 (26.8; 26.8) | 3626 (27.6; 30.0) | 3612 (27.5; 30.8) | 3904 (29.8; 34.5) | 3887 (29.6; 35.9) | 3799 (29.0; 37.2) |
|  | *Recent* | 2995 (22.8; 22.8) | 2434 (18.6; 20.1) | 2284 (17.4; 19.5) | 1782 (13.6; 15.8) | 1565 (11.9; 14.5) | 1327 (10.1; 13.0) |
| **Duration of smoking (cumul.; years)**  **(median; IQR)** | *Former* | 12.0(7.0, 19.0) | 13.0(7.0, 22.0) | 14.0(7.0, 23.0) | 15.0(8.0, 25.0) | 15.5(8.0, 26.0) | 16.0(8.0, 27.5) |
|  | *Recent* | 26.5(21.5, 32.0) | 30.5(25.5, 36.0) | 32.5(27.0, 38.5) | 35.5(30.5, 41.0) | 38.5(33.0, 43.5) | 41.0(35.5, 46.0) |
| **Duration since quitting (cumul.; years) (median; IQR)** | *Former* | 15.0(9.0, 21.5) | 18.5(11.5, 26.0) | 20.5(12.5, 28.5) | 22.5(13.0, 30.5) | 25.0(14.5, 33.5) | 27.0(16.0, 35.5) |
|  | *Recent* | 0.0(0.0, 0.0) | 0.0(0.0, 0.0) | 0.0(0.0, 1.0) | 0.0(0.0, 0.0) | 0.0(0.0, 1.0) | 0.0(0.0, 1.5) |
| **Pack years (cumul.)**  **(median; IQR)** | *Former* | 4.6(1.8, 10.0) | 5.2(2.0, 11.2) | 5.5(2.0, 11.8) | 6.0(2.1, 12.9) | 6.1(2.1, 13.1) | 6.4(2.2, 13.9) |
|  | *Recent* | 14.6(8.0, 22.4) | 17.3(9.6, 26.2) | 18.7(10.0, 27.9) | 21.4(12.2, 30.8) | 22.9(13.5, 33.0) | 24.1(14.3, 34.3) |
| **Lung cancer screening criteria**  **for smokers (N (%))** | *met* | 1232 (9.4; 18.9 **^b^**) | 1528 (11.6; 25.2) | 1555 (11.9; 26.4) | 1505 (11.5; 26.5) | 1377 (10.5; 25.3) | 1224 (9.3; 23.9) |
|  | *not met* | 5280 (40.2; 81.1) | 4532 (34.5; 74.8) | 4341 (33.1; 73.6) | 4181 (31.9; 73.5) | 4075 (31.1; 74.7) | 3902 (29.7; 76.1) |
| **Deaths ^c^ (N (%))** | | 171(1.3; 16.5 **^d^**) | 250(1.9; 18.0) | 357(2.7; 19.7) | 497(3.8; 21.7) | 637(4.9; 21.9) | 1028(7.8) |
| *Deaths from lung cancer (N (%))* | | 9(0.1; 0.9) | 13(0.1; 0.9) | 20(0.2; 1.1) | 34(0.3; 1.5) | 48(0.4; 1.7) | 75(0.6) |
| *Deaths from other causes (N (%))* | | 162(1.2; 15.6) | 237(1.8; 17.0) | 337(2.6; 18.6) | 463(3.5; 20.2) | 589(4.5; 20.3) | 953(7.3) |
| **Lost to follow up (N (%))** | | 865(6.6; 83.5) | 1141(8.7; 82.0) | 1458(11.1; 80.3) | 1798(13.7; 78.3) | 2268(17.3; 78.1) |  |

1. Two percentages are computed for discrete variables. The denominator for the first percentage is the total number of female participants (13, 121), while the denominator for the second percentage is the number of female individuals who actually participated at the follow-up round.
2. The denominator for the second percentage is the number of ever female smokers (include former smoker and recent smoker) who actually participated the follow-up round.
3. The right censoring time was set to the expected date of the next follow-up visit — 6 years after baseline and 3 years after the second to fifth visits. For participants who completed the last (sixth) follow-up round, the censoring time was defined as 5 years after that visit.

Participants who died before the censoring time were considered to have experienced the event, whereas those who were permanently lost to follow-up were right-censored.

1. The denominator of the second percentage is the total number of female participants who either died (event) or were censored due to loss to follow-up up to the expected censoring time.

**Table S2: Hazard ratios and P values for lung cancer incidence risks by smoking-related continuous variables in the EPIC-Heidelberg cohort**

|  | **Cox model for men (n= 11594)** | | **Cox model for women (n= 13121)** | |
| --- | --- | --- | --- | --- |
|  | **Hazard ratio (95% CI)** | **P value** | **Hazard ratio (95% CI)** | **P value** |
| **Lung cancer incidence** | (event number = 180) |  | (event number = 81) |  |
| Duration of smoking (per 5 yrs.) | 1.35 (1.25 - 1.45) | < 0.001 | 1.19 (1.11 - 1.27) | < 0.001 |
| Duration since quitting (per 5 yrs.) | 1.02 (0.93 - 1.12) | 0.68 | 1.04 (0.94 - 1.15) | 0.47 |
| Daily Cigarette Consumption  (per 10 Cigarettes/Day) | 1.44 (1.31 - 1.58) | < 0.001 | 1.76 (1.45 - 2.14) | < 0.001 |

- The right censoring time was set to the expected date of the next follow-up visit — 6 years after baseline and 3 years after the second to fifth visits. For participants who completed the last (sixth) follow-up round, the censoring time was defined as 5 years after that visit.

Participants who were diagnosed by lung cancer before the censoring time were considered to have experienced the event, whereas those who were permanently lost to follow-up were right-censored.

- Time-varying Cox regression models were employed to evaluate the association of smoking-related continuous variables on lung cancer incidence risks, using age as underlying time scale and with mutual adjustment between the smoking-related variables (but no further adjustments).

**
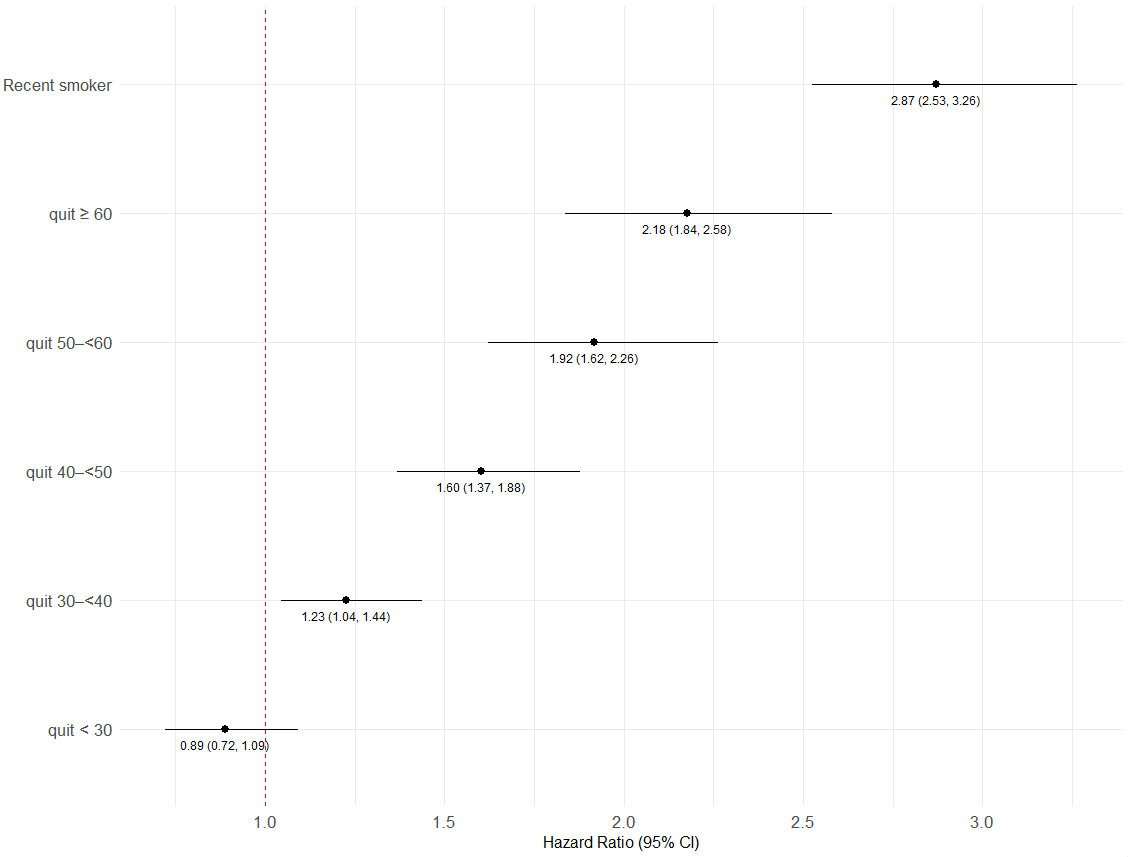
**

**Figure S1: (A) Hazard ratios for all-cause mortality by sex and age-at-quitting categories in the EPIC-Heidelberg cohort (men)**

- Hazard ratios were estimated relative to never smokers as reference category, using a model with age as underlying time scale and without any further adjustments.

**
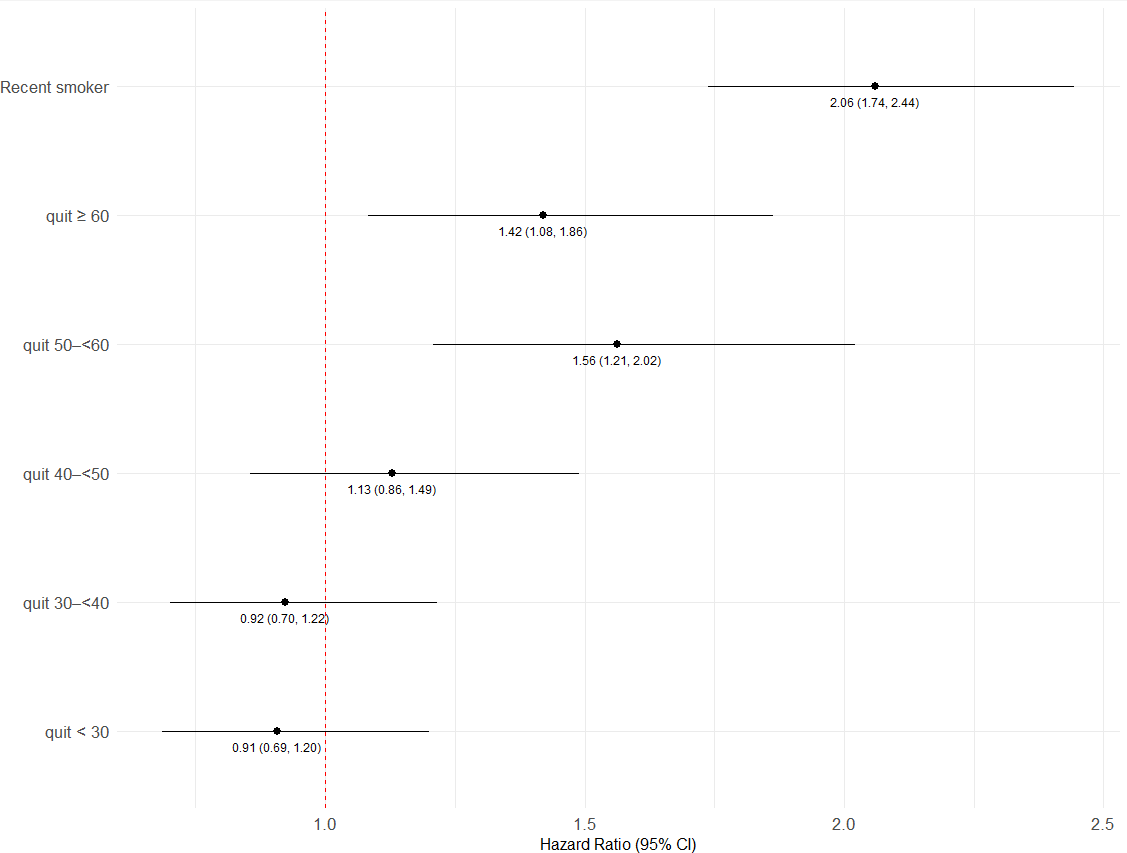
**

**Figure S1: (B) Hazard ratios for all-cause mortality by sex and age-at-quitting categories in the EPIC-Heidelberg cohort (women)**

- Hazard ratios were estimated relative to never smokers as reference category, using a model with age as underlying time scale and without any further adjustments.
